# Atherogenic index of plasma and severe headaches or migraines risk in US adults: a population-based cross-sectional analysis from NHANES 1999–2004

**DOI:** 10.3389/fneur.2026.1665147

**Published:** 2026-05-26

**Authors:** Shaoxiong Chen, Yi He, Jiaheng Xu

**Affiliations:** 1 Department of Neurosurgery, the First Affiliated Hospital, Fujian Medical University, Fuzhou, Fujian, China; 2 Department of Neurosurgery, National Regional Medical Center, Binhai Campus of the First Affiliated Hospital, Fujian Medical University, Fuzhou, China

**Keywords:** atherogenic index of plasma, high-density lipoprotein, cholesterol, national health and nutrition examination survey, severe headaches or migraines, triglycerides

## Abstract

**Background:**

Despite the established link between the atherogenic index of plasma (AIP) and cardiometabolic diseases, its association with severe headaches or migraines remains poorly characterized. This study aimed to explore the association between AIP and severe headaches or migraines using data from the National Health and Nutrition Examination Survey (NHANES).

**Methods:**

Data were drawn from the NHANES 1999–2004, comprising 6,017 adults. AIP was calculated as log10 [triglycerides/high-density lipoprotein cholesterol (HDL-C)] and stratified into quartiles. Severe headaches or migraines status was identified through self-reported responses. Survey-weighted multivariable logistic regression was used to evaluate the association between AIP and the risk of severe headaches or migraines, with restricted cubic spline (RCS) models employed to characterize the dose-response relationship. Subgroup and sensitivity analyses were performed to assess consistency and robustness.

**Results:**

Among 1,218 severe headache or migraine patients, baseline characteristics revealed that severe headache or migraine patients were predominantly younger (<45 years: 57.6%), female (57.6%), and had higher smoking rates (56.6%). Higher risk of severe headaches or migraines was observed in the second (Q2: 26.5%) and fourth quartiles (Q4: 27.2%) of AIP. After full adjustment, Q2 (*OR* = 1.481, 95% CI: 1.105–1.984) and Q4 (*OR* = 1.799, 95% CI: 1.228–2.637) exhibited significantly elevated severe headaches or migraines risks compared to Q1. RCS analysis demonstrated a significant overall association between AIP and severe headaches or migraines (*p* = 0.029), though a definitive non-linear trend was not statistically confirmed. Subgroup analyses emphasized that individuals in the age <45 years (*OR* = 1.50), female (*OR* = 1.47), and no diabetes (*OR* = 1.31) or coronary heart disease (*OR* = 1.26; *p* < 0.05) subgroups were at a higher risk for severe headaches or migraines (*p* < 0.05). The eXtreme Gradient Boosting (XGBoost) modeling identified AIP (19.34%) as a highly influential feature associated with severe headaches or migraines.

**Conclusion:**

Elevated AIP was associated with self-reported severe headaches or migraines in this cross-sectional analysis, though a consistent dose-response relationship was not established. These findings suggest a potential link with lipid metabolism, but causality cannot be inferred. Prospective studies are required to further evaluate this association.

## 1 Introduction

Migraine, a chronic central nervous system disorder with high global prevalence and significant disability burden, affects approximately 15% of the population and remains the leading cause of disability among individuals aged 15–49 years (1, 2). A substantial sex disparity exists, with women experiencing migraine at nearly three times the rate of men, particularly during hormonally sensitive periods such as menstruation, pregnancy, and menopause. Despite a broad spectrum of therapeutic approaches, including acute abortive medications, preventive pharmacotherapy, and non-pharmacological strategies such as dietary and sleep regulation, the precise pathophysiological mechanisms underlying migraine remain inadequately defined (3, 4). In this study, we focus on self-reported severe headaches or migraines as the outcome, as ascertained by a standard NHANES questionnaire item.

A substantial knowledge gap persists regarding the contribution of metabolic dysregulation to severe headaches or migraines susceptibility and progression. Although stratified acute treatment based on symptom severity and comorbidities is commonly adopted, and preventive treatment is estimated to benefit approximately 38% of patients with episodic severe headaches or migraines, real-world utilization remains suboptimal, with fewer than 13% of eligible individuals receiving prophylactic medications (3, 5, 6). Metabolic disturbances, such as dyslipidemia and insulin resistance, are hypothesized to contribute to severe headaches or migraines pathogenesis by promoting neuroinflammation, endothelial dysfunction, and cortical spreading depression, ultimately activating the trigeminovascular system (7, 8). Despite these advances, a specific biomarker that can integratively reflect such metabolic alterations and their connection to severe headaches or migraines risk remains to be identified.

The atherogenic index of plasma (AIP), calculated as log10 [triglycerides (TG)/high-density lipoprotein cholesterol (HDL-C)], serves as a robust composite indicator reflecting the atherogenic potential of plasma lipids and a surrogate marker for insulin resistance. Compared with individual lipid parameters, AIP more sensitively captures early lipid metabolic imbalance and chronic low-grade inflammation, both of which are mechanistically relevant to severe headaches or migraines (9–12). Notably, severe headaches or migraines patients often exhibit a lipid profile akin to that of elevated AIP (characterized by high TG and low HDL-C), suggesting potential shared pathways involving oxidative stress, endothelial injury, and neuroinflammatory activation (13, 14). Therefore, AIP may represent a biologically meaningful and easily obtainable metabolic biomarker for assessing severe headaches or migraines risk. It offers practical advantages for large-scale population studies due to its simplicity, cost-effectiveness, and established clinical validity in metabolic and cardiovascular diseases. Nevertheless, no population-based studies have systematically evaluated the association between AIP and severe headaches or migraines risk.

This study leverages nationally representative clinical and biochemical data from the National Health and Nutrition Examination Survey (NHANES) to address this research gap. It was hypothesized that elevated AIP would be associated with increased risk of severe headaches or migraines. By elucidating potential links between this emerging metabolic biomarker and severe headaches or migraines, the findings aim to advance understanding of the metabolic underpinnings of severe headaches or migraines susceptibility and inform novel, evidence-based preventive strategies (15).

## 2 Methods

### 2.1 Data source and study population

A cross-sectional analysis was conducted utilizing data from the NHANES 1999–2004 to investigate the association between the AIP and the risk of severe headaches or migraines (16, 17). NHANES, administered by the US Centers for Disease Control and Prevention (CDC), employs a complex, stratified, multistage probability sampling design, enabling nationally representative estimates for the non-institutionalized US population. Following the NCHS guidelines, we pooled three survey cycles and created a composite mobile examination center (MEC) weight (WTMEC11YR) by dividing the original 2-year MEC weights (WTMEC2YR) by the number of cycles (i.e., three). A survey design object was then constructed in R using the survey package, incorporating the adjusted weight, strata (SDMVSTRA), and primary sampling units (SDMVPSU). Data collection and reporting strictly adhered to the Strengthening the Reporting of Observational Studies in Epidemiology (STROBE) guidelines.

**Figure 1:**
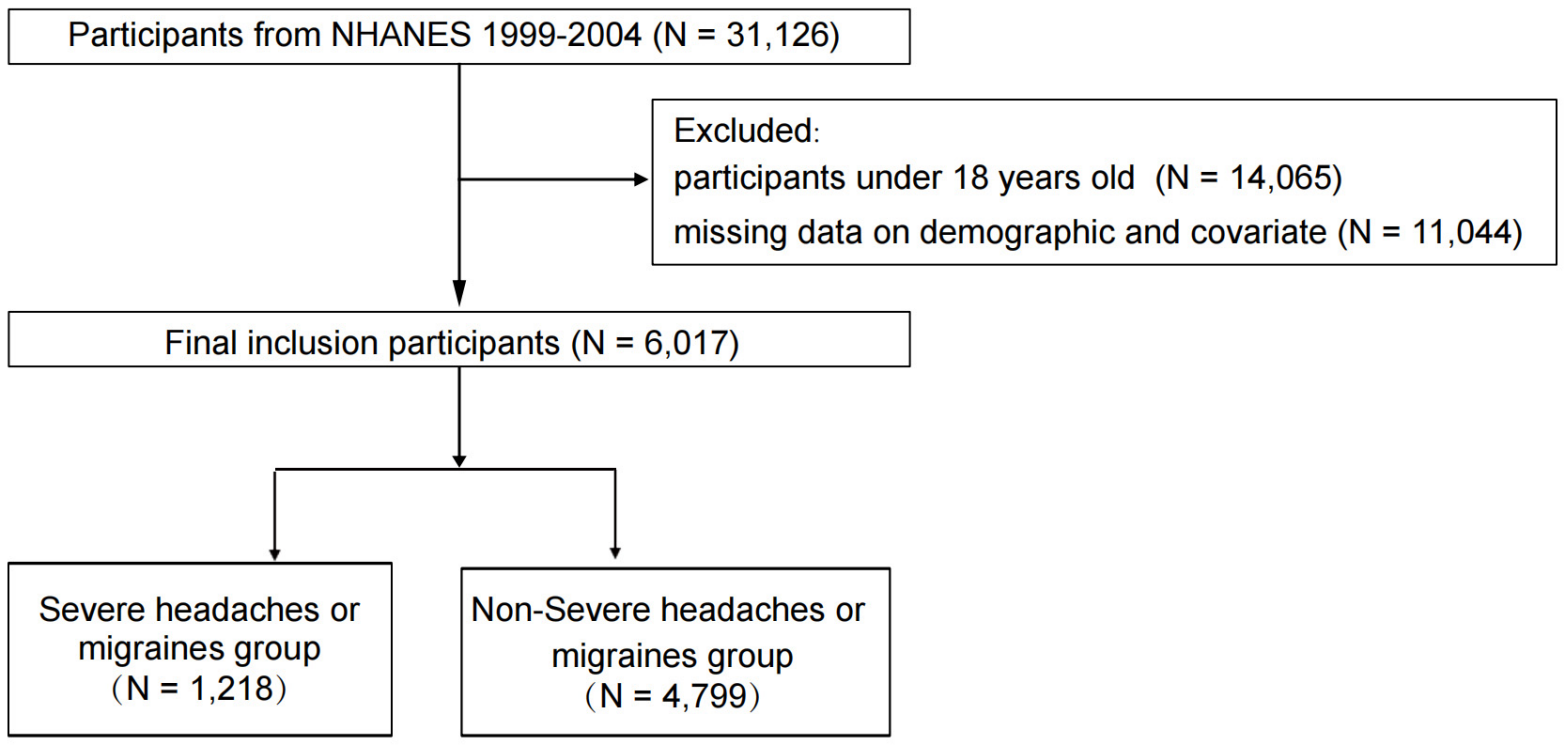
Flow chart of participant selection for the study of severe headaches or migraines.

Among 31,126 participants enrolled during the study period, individuals with missing data on severe headaches or migraine status, key variables, or essential covariates (e.g., age, sex, smoking status) were excluded. In addition, 14,065 participants aged younger than 18 years were excluded, followed by sequential exclusions due to missing data for diabetes (215), hypertension (235), smoking status (9,402), educational level (16), marital status (254), severe headaches or migraines (3), stroke (9), coronary heart disease (CHD, 53), HDL-C (840), and TG (17). The final analytic sample included 6,017 participants (Figure 1). NHANES-provided sampling weights were incorporated to account for survey design complexity and non-response, ensuring national representativeness. Written informed consent was obtained from all participants, and the study protocol was approved by the National Center for Health Statistics Research Ethics Review Board (18, 19).

### 2.2 Exposure and outcome variables

The exposure variable, AIP, was calculated as the base-10 logarithm of the ratio of TG to HDL-C, expressed as *AIP* = log_10_(*TG*/*HDL*−*C*). Serum lipid levels were measured using standardized CDC laboratory protocols, including direct immunoassay or precipitation methods. Fasting venous blood samples were collected for TG measurement. Participants were stratified into quartiles based on AIP values for further analysis (20).

Severe headaches or migraines status was determined based on self-reported responses to the Miscellaneous Pain Questionnaire (MPQ090): “During the past 3 months, have you had severe headaches or migraines?” A positive response was classified as severe headaches or migraines (21). This self-reporting approach has been repeatedly used as a dependable outcome measure in well-established NHANES-based severe headaches or migraines literature (22) and shows moderate-to-high agreement with clinician-based diagnosis (23).

### 2.3 Covariates

Covariates were selected based on prior literature and clinical relevance, including age, sex (male/female), marital status (married/unmarried), education level (less than high school, high school, more than high school), smoking status, hypertension, diabetes, stroke, and CHD. Age was further categorized into < 45, 45–64, and ≥65 years for subgroup analyses (Supplementary Table S1).

### 2.4 Statistical analysis

All analyses accounted for the complex survey design and incorporated sample weights. Continuous variables were described using weighted means and standard deviations (SDs) or medians with interquartile ranges (IQRs), while categorical variables were summarized using frequencies and percentages. Group comparisons were performed using analysis of variance (ANOVA) for normally distributed variables, Kruskal–Wallis tests for skewed distributions, or chi-square tests for categorical variables (24).

Survey-weighted multivariable logistic regression was used to estimate odds ratios (ORs) and 95% confidence intervals (CIs) for the association between AIP and severe headaches or migraines (25). Model 1 was unadjusted, Model 2 adjusted for age, sex, marital status, and education, Model 3 further adjusted for hypertension, diabetes, stroke, smoking status, and CHD. Potential non-linear associations were explored using restricted cubic spline (RCS) regression with 4 knots to model the exposure, a choice made to optimally capture potential non-linearity while preventing overfitting (26).

Subgroup analyses were stratified by age, sex, marital status, education, and comorbidities. Sensitivity analyses using unweighted logistic regression models were conducted to assess the robustness of results.

To evaluate the relative importance of variables associated with severe headaches or migraine risk, the eXtreme Gradient Boosting (XGBoost) algorithm was applied (27). Model hyperparameters, including a learning rate of 0.1, maximum tree depth of 5, and 500 boosting rounds, were optimized through 10-fold cross-validation to minimize overfitting. Feature importance was quantified using the average gain metric, which reflects the contribution of each variable to model accuracy across decision trees. Continuous covariates were standardized (mean = 0, *SD* = 1). The analysis focused on identifying the most influential features associated with severe headaches or migraines, with results interpreted alongside traditional regression models to enhance robustness and clinical relevance.

All analyses were performed using R version 4.4.2, and all tests were two-tailed with a significance level set at *p* < 0.05.

## 3 Result

### 3.1 Baseline characteristics

A total of 6,017 participants were included in the analysis, comprising 1,218 individuals with severe headaches or migraines and 4,799 without. Significant differences were observed between groups regarding age, sex, marital status, education, hypertension, smoking status, CHD, HDL-C levels, and AIP (all *p* < 0.05; Table 1). Participants with severe headaches or migraines were predominantly younger (< 45 years, 57.6%), female (57.6%), married (53.5%), had educational attainment below high school (36.7%), and reported higher rates of smoking (56.6%). Compared with the severe headaches or migraines-free group, participants with severe headaches or migraines exhibited a distinct lipid profile; TG levels were slightly higher (1.79 ± 1.46 vs. 1.74 ± 1.83 mg/dl), whereas HDL-C levels were lower (1.31 ± 0.41 vs. 1.34 ± 0.42 mg/dl). A greater proportion of severe headaches or migraines fell into the highest quartile (Q4) of the atherogenic index of plasma (27.2 %), while the non-severe headaches or migraines group had a higher representation in the lowest quartile (Q1, 25.7 %).

**Table 1:** Baseline characteristics of the study population based on AIP.

Variable	Level	Yes	No	*p*
N		1,218	4,799
Age (%)	< 45	701 (57.6)	1,752 (36.5)	< 0.001
	45–64	379 (31.1)	1,645 (34.3)	
	≥65	138 (11.3)	1,402 (29.2)	
Sex (%)	Male	517 (42.4)	2,964 (61.8)	< 0.001
	Female	701 (57.6)	1,835 (38.2)	
Marital (%)	Married	652 (53.5)	3,246 (67.6)	< 0.001
	Unmarried	566 (46.5)	1,553 (32.4)	
Educational (%)	Below high school	447 (36.7)	1,587 (33.1)	0.008
	Senior high school	330 (27.1)	1,246 (26.0)	
	Above high school	441 (36.2)	1,966 (41.0)	
Hypertension (%)	Yes	377 (31.0)	1,631 (34.0)	0.049
	No	841 (69.0)	3,168 (66.0)	
Diabetes (%)	Yes	120 (9.9)	531 (11.1)	0.244
	No	1,098 (90.1)	4,268 (88.9)	
Stroke (%)	Yes	53 (4.4)	172 (3.6)	0.24
	No	1,165 (95.6)	4,627 (96.4)	
Smoking status (%)	Yes	689 (56.6)	2,018 (42.1)	< 0.001
	No	529 (43.4)	2,781 (57.9)	
Coronary heart disease (%)	Yes	48 (3.9)	314 (6.5)	0.001
	No	1,170 (96.1)	4,485 (93.5)	
HDL-C [mean (*SD*)]		1.31 (0.41)	1.34 (0.42)	0.048
TG [mean (*SD*)]		1.79 (1.46)	1.74 (1.83)	0.44
AIP (%)	Q1	273 (22.4)	1,232 (25.7)	0.027
	Q2	323 (26.5)	1,181 (24.6)	
	Q3	291 (23.9)	1,213 (25.3)	
	Q4	331 (27.2)	1,173 (24.4)	

HDL-C, high-density lipoprotein cholesterol; TG, triglycerides; AIP, atherogenic index of plasma.

### 3.2 Association between AIP and severe headaches or migraines

Survey-weighted logistic regression revealed that higher AIP levels were associated with an increased risk of severe headaches or migraines. In the unadjusted model (Model 1), participants in the Q2 (*OR* = 1.345, 95% CI: 1.070–1.690, *p* = 0.012) and Q4 (*OR* = 1.384, 95% CI: 1.122–1.709, *p* = 0.003) exhibited significantly higher risk of severe headaches or migraines compared with those in the lowest quartile. After adjusting for Model 2, ORs increased to 1.517 (95% CI: 1.195–1.926, *p* = 0.001) for Q2, 1.432 (95% CI: 1.096–1.872, *p* = 0.010) for the third quartile (Q3), and 1.802 (95% CI: 1.409–2.304, *p* < 0.001) for Q4, respectively. In the fully adjusted model (Model 3), compared with the lowest quartile, the ORs for Q2, Q3, and Q4 were 1.501 (*p* = 0.002), 1.376 (*p* = 0.027), and 1.711 (*p* < 0.001), respectively These findings indicated that elevated AIP was a significant risk factor for severe headaches or migraines, with a stronger effect observed in higher exposure groups (Table 2). The multicollinearity diagnostics for the fully adjusted model (Model 3) confirmed the absence of severe collinearity, with all variance inflation factors (VIF) values ranging between 1.02 and 1.40 (Supplementary Table S2). Specifically, the VIF for AIP was 1.19, for TG was 1.30, and for HDL-C was 1.40. This supports the robustness of the observed association between AIP and severe headaches or migraines.

**Table 2:** Logistic regression analysis of AIP and severe headaches or migraines.

Exposure	Model 1 *OR* (95% CI)	*p*	Model 2 *OR* (95% CI)	*p*	Model 3 *OR* (95% CI)	*p*
Q1	Ref	Ref	Ref	Ref	Ref	Ref
Q2	1.345 (1.070–1.690)	0.012	1.517 (1.195–1.926)	0.001	1.501 (1.179–1.911)	0.002
Q3	1.178 (0.918–1.512)	0.191	1.432 (1.096–1.872)	0.010	1.376 (1.040–1.822)	0.027
Q4	1.384 (1.122–1.709)	0.003	1.802 (1.409–2.304)	< 0.001	1.711 (1.322–2.213)	< 0.001

OR: odds ratio.

Model 1 was unadjusted.

Model 2 adjusted for age, sex, marital status, and educational level.

Model 3 further adjusted for hypertension, diabetes, stroke, smoking status, and coronary heart disease.

**Figure 2:**
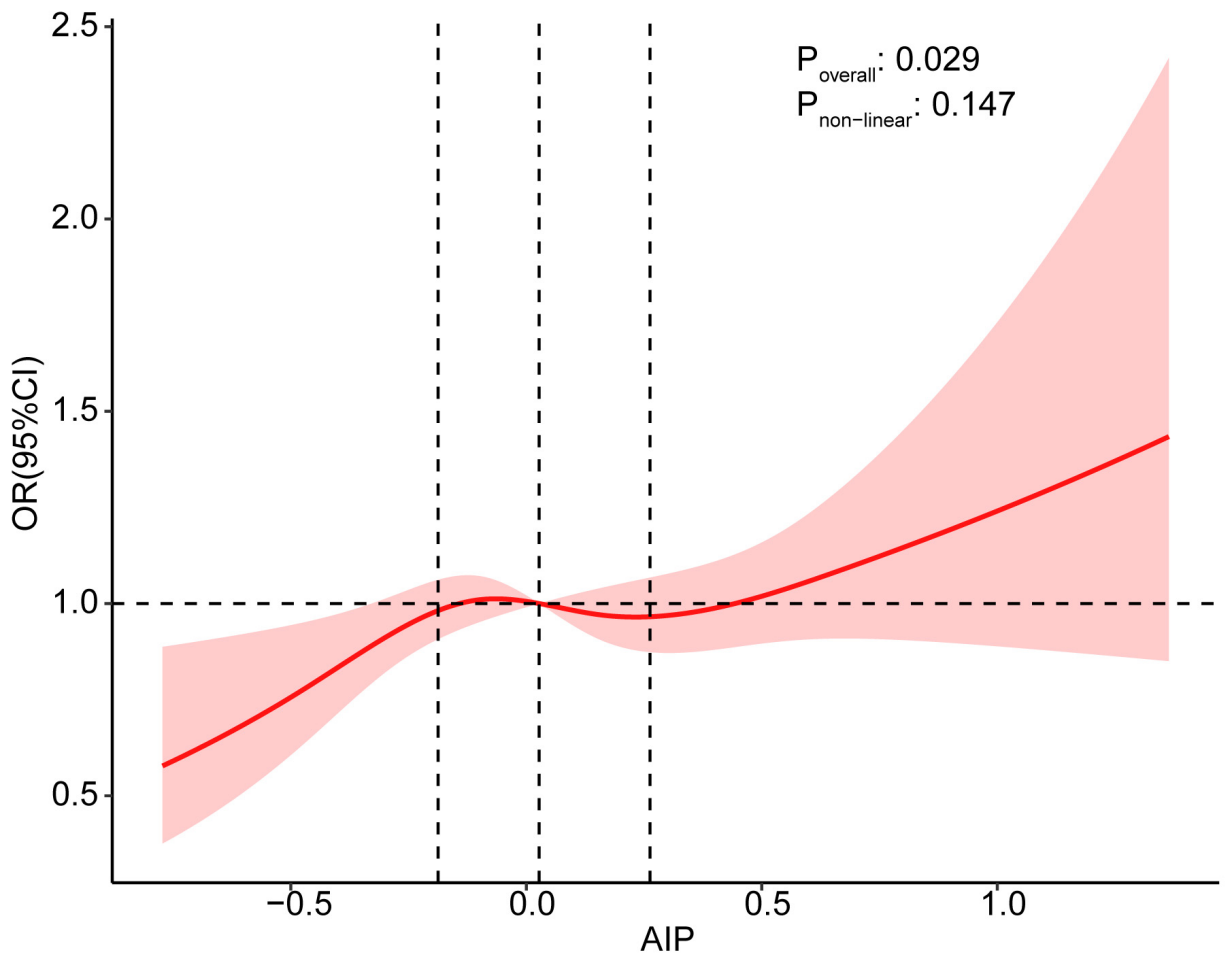
Restricted cubic spline curves for the association between AIP and severe headaches or migraines.

RCS analysis demonstrated a significant overall association between AIP and severe headaches or migraines risk (*P*_overall_ = 0.029). However, the test for non-linearity did not reach statistical significance (*P*_non − linear_ = 0.147), suggesting that while risk generally increases with AIP, the dose-response curve may exhibit instability or follow a modest linear trend rather than a definitive non-linear trajectory (Figure 2).

### 3.3 Subgroup analyses

**Figure 3:**
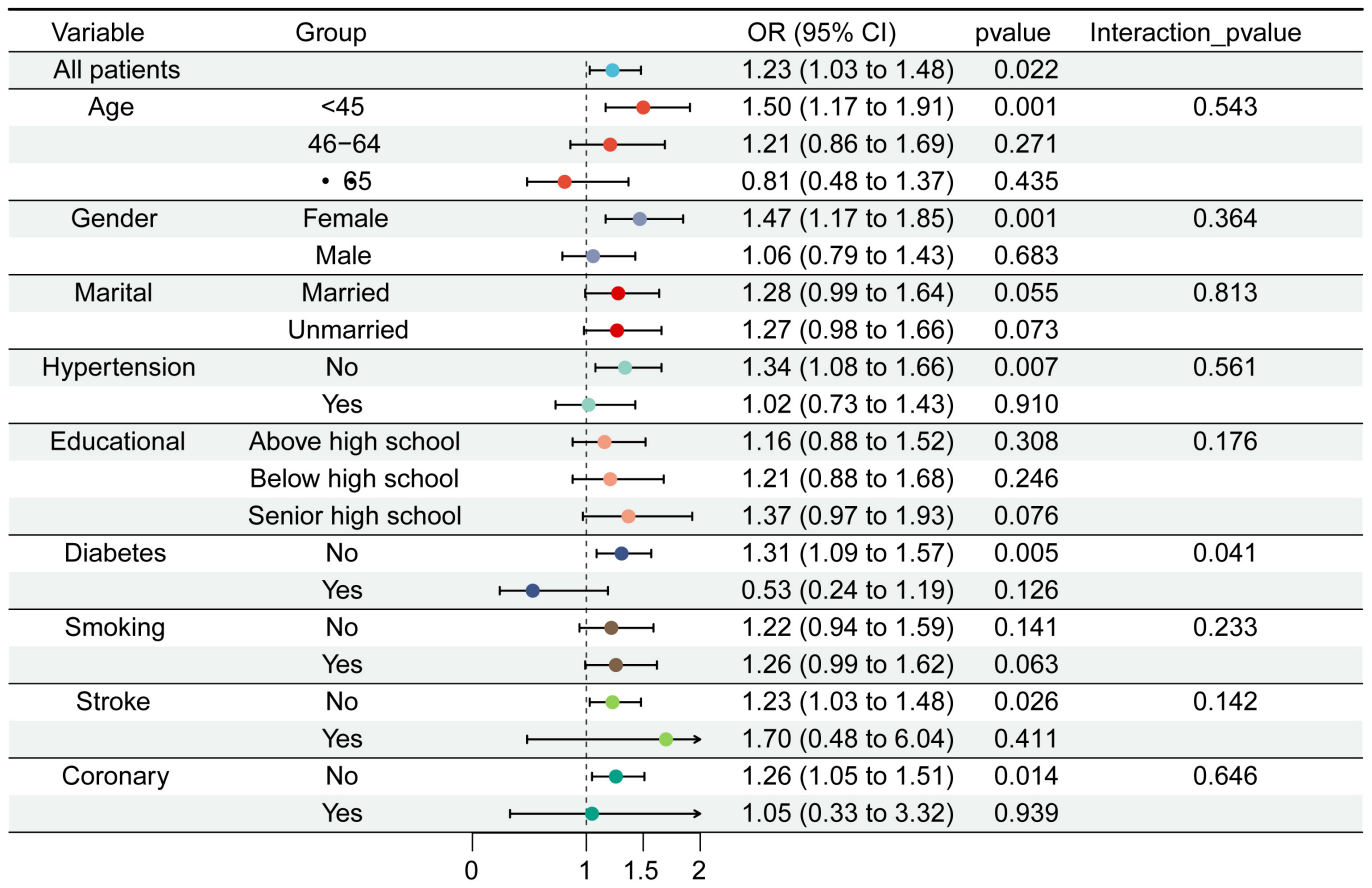
Forest plot of subgroup analysis of the association of AIP and severe headaches or migraines.

Subgroup analyses revealed consistent positive associations between AIP and severe headaches or migraines across multiple strata. Higher risk for severe headaches or migraines were observed among individuals aged < 45 years (*OR* = 1.50, 95% CI: 1.17–1.91, *p* = 0.001), females (*OR* = 1.47, 95% CI: 1.17–1.85, *p* = 0.001), and those without hypertension (*OR* = 1.34, 95% CI: 1.08–1.66, *p* = 0.007), diabetes (*OR* = 1.31, 95% CI: 1.09–1.57, *p* = 0.005), stroke (*OR* = 1.23, 95% CI: 1.03–1.48, *p* = 0.026), or CHD (*OR* = 1.26, 95% CI: 1.05–1.51, *p* = 0.014). No significant interactions were observed for most stratifications (*p* for interaction >0.05), except for diabetes (*p* for interaction = 0.041). People without diabetes have a higher risk of severe headaches or migraines (*OR* = 1.31, *p* = 0.005; Figure 3).

**Figure 4:**
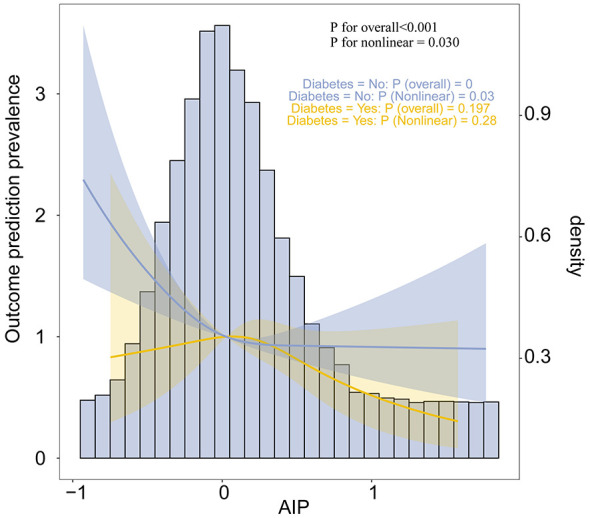
Association between AIP and severe headaches or migraines stratified by factor diabetes mellitus.

The subgroup analysis unveiled a significant interaction effect of diabetes on the association between AIP and the risk of severe headaches or migraines. To further disentangle this complex relationship and explore potential non-linear patterns, RCS analysis was applied (Figure 4). In the non-diabetic population, an increase in AIP was associated with a higher risk of severe headaches or migraines (*p*_overall_ = 0, *p*_non − linear_ = 0.03).

### 3.4 Sensitivity analysis of AIP and severe headaches or migraines risk

In the sensitivity analyses examining the relationship between AIP and severe headaches or migraines risk, unweighted logistic regression models were used to assess the relationship between different exposure levels (Q1–Q4). The logistic regression model showed a statistically significant positive association between higher AIP exposure levels and the risk of severe headaches or migraines (Table 3).

**Table 3:** Unweighted logistic regression model analysis of AIP with severe headaches or migraines.

Exposure	Model 1 *OR* (95%CI)	*P*-value	Model 2 *OR* (95%CI)	*P*-value	Model 3 *OR* (95%CI)	*P*-value
Q2	1.234 (1.0314–1.478)	0.022	1.367 (1.134–1.648)	0.001	1.351 (1.121–1.630)	0.002
Q3	1.083 (0.9015–1.301)	0.396	1.283 (1.060–1.555)	0.011	1.253 (1.033–1.519)	0.022
Q4	1.273 (1.0649–1.524)	0.008	1.637 (1.354–1.981)	< 0.001	1.571 (1.296–1.907)	< 0.001

OR, odds ratio.

Model 1 was unadjusted.

Model 2 adjusted for age, sex, marital status, and educational level.

Model 3 further adjusted for hypertension, diabetes, stroke, smoking status, and coronary heart disease.

### 3.5 XGBoost analysis

**Figure 5:**
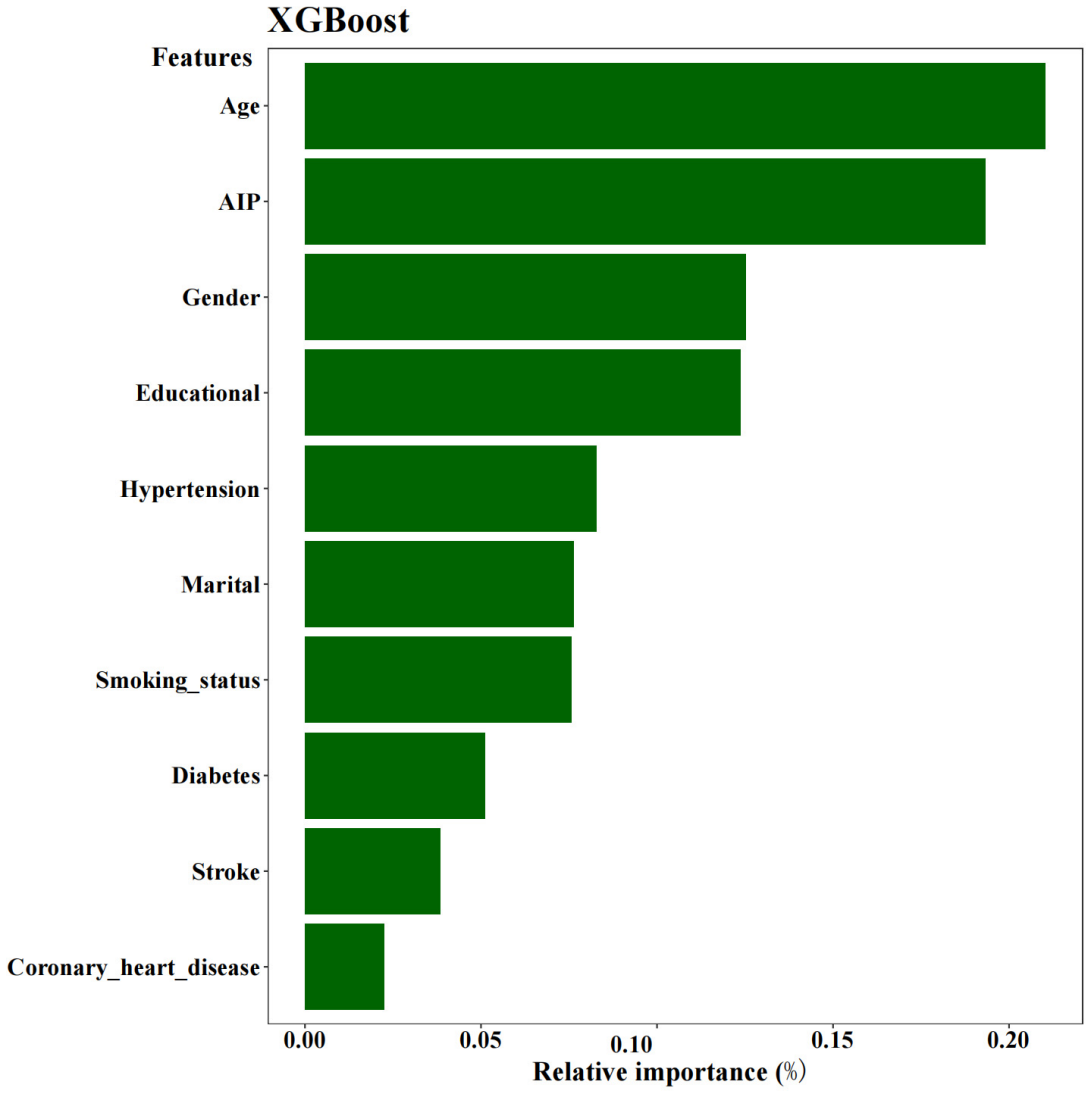
Relative importance of variables in the XGBoost model for severe headaches or migraines prediction.

The XGBoost feature importance analysis identified the relative contributions of key predictors for severe headaches or migraines. Age emerged as the most influential variable with a contribution of 21.04%, followed closely by the AIP at 19.34%. Other significant contributors included gender (12.53%) and educational level (12.38%). Interestingly, AIP showed a high predictive weight that was almost equal to age, underscoring its crucial function as a metabolic biomarker for excruciating migraines or headaches (Figure 5).

## 4 Discussion

Analysis of nationally representative NHANES data identified a modest positive association between AIP and self-reported severe headaches or migraines. This relationship was more pronounced among individuals under 45 years of age, women, and those without major metabolic comorbidities (e.g., diabetes, coronary heart disease). These findings provide suggestive evidence for a potential link between lipid metabolism and severe headaches or migraines.

Our results are broadly consistent with prior studies linking traditional lipid metrics to severe headaches or migraines susceptibility (28). AIP, as a log-transformed ratio of triglycerides to HDL-C, offers an integrated measure of atherogenic dyslipidemia. Its association with self-reported severe headaches or migraines in this cross-sectional sample suggests that further investigation into lipid-related pathways may be warranted.

The stronger association observed among women and younger individuals warrants cautious interpretation. Some literature suggests that estrogens modulate lipid metabolism (29, 30), and we noted that our findings regarding the stronger association between AIP and migraine in women align with recent research on population-specific risk profiles (e.g., Martini et al.). These studies also underscore the necessity of identifying unique risk characteristics and relevant comorbidities, such as sleep disorders, when managing migraine in female populations (31, 32). But the cross-sectional design of this study cannot determine temporal or causal relationships. Notably, AIP remained a significant correlate even in individuals without diabetes or hypertension, suggesting that lipid imbalance may precede metabolic syndrome. However, this hypothesis requires prospective validation.

Several limitations of this study warrant consideration. First, due to the cross-sectional design, the temporal sequence between AIP levels and the onset of severe headaches or migraines remains indeterminate, rendering the findings associative rather than causal. Second, severe headaches or migraines status was ascertained by self-report, while this method shows moderate-to-high agreement with clinical diagnoses, any non-differential misclassification would likely bias our results toward the null, leading to an underestimation of the true association. Third, while we adjusted for major potential confounders, residual confounding by unmeasured variables (e.g., detailed dietary patterns, physical activity levels, or genetic predisposition) cannot be excluded. Fourth, the lack of data on lipoprotein subfractions (e.g., small dense LDL particles or oxidized HDL) and specific inflammatory markers limits our ability to fully characterize the underlying biological mechanisms. Future prospective cohorts with serial metabolic measurements and clinically confirmed severe headaches or migraine diagnoses are needed to verify causality and minimize bias.

## 5 Conclusion

In summary, this cross-sectional analysis found a modest positive association between elevated AIP and self-reported severe headaches or migraines, particularly among younger, female, and metabolically healthier subgroups. These findings suggest a potential link between adverse lipid metabolism and this headache phenotype. However, due to the cross-sectional design and the self-reported outcome, causality cannot be inferred. Our results are hypothesis-generating and require confirmation in prospective studies with clinically diagnosed outcomes.

## Data Availability

The original contributions presented in the study are included in the article/Supplementary material, further inquiries can be directed to the corresponding author.
