# Supplementary material for: Atherogenic index of plasma and severe headaches or migraines risk in US adults: a population-based cross-sectional analysis from NHANES 1999–2004
Source: Front Neurol. 2026 May 26;17:1665147. doi: 10.3389/fneur.2026.1665147 (PMC13246651; doi:10.3389/fneur.2026.1665147)
Supplement: Supplementary file 1 [file Table_1.docx]

Table S1 Summary of covariates from the NHANES 1999-2004.

| Variable | Code | Definition | Year |
| --- | --- | --- | --- |
| Age | RIDAGEYR | Respondent's best age in years at the time of the household interview. Persons aged 85 and older are coded as 85 years old. | 1999-2004 |
| Gender | RIAGENDR | Male or Female | 1999-2004 |
| Educational Level | DMDHREDU | What is the highest grade or year of school completed by you/NON_SP HEAD? | 1999-2004 |
| Smoking | SMQ040 | Do you smoke now? | 1999-2004 |
| Marital Status | DMDHRMAR | Marital status of the participant | 1999-2004 |
| Triglycerides | LBDSTRSI | Triglycerides (mmol/L) | 1999-2004 |
| HDL-C | LBDHDD/LBDHDDSI | DirectHDL-Cholesterol(mg/dL)  DirectHDL-Cholesterol(mmol/L) | 1999-2004 |
| Hypertension | BPQ020 | {Have you/Has SP} ever been told by a doctor or other health professional that {you/s/he} had hypertension, also called high blood pressure? | 1999-2004 |
| Diabetes | DIQ010 | Doctor-diagnosed diabetes | 1999-2004 |
| Stroke | MCQ160F | Has a doctor or other health professional ever told {you/SP} that {you/s/he} had a stroke? | 1999-2004 |
| Coronary Heart Disease | MCQ160C | Has a doctor or other health professional ever told {you/SP} that {you/s/he} had coronary heart disease? | 1999-2004 |

Table S2. Multicollinearity diagnostics for variables in the fully adjusted logistic regression model (Model 3).

| Variable | VIF | Tolerance | Collinearity Status |
| --- | --- | --- | --- |
| AIP | 1.186 | 0.843 | Acceptable |
| Age | 1.1 | 0.909 | Acceptable |
| Gender | 1.052 | 0.951 | Acceptable |
| Marital | 1.073 | 0.932 | Acceptable |
| Educational | 1.016 | 0.984 | Acceptable |
| Hypertension | 1.105 | 0.905 | Acceptable |
| Diabetes | 1.062 | 0.942 | Acceptable |
| Stroke | 1.037 | 0.965 | Acceptable |
| Smoking status | 1.095 | 0.913 | Acceptable |
| Coronary heart disease | 1.042 | 0.959 | Acceptable |
| HDL-C | 1.4 | 0.714 | Acceptable |
| TG | 1.299 | 0.77 | Acceptable |

Note: VIF: variance inflation factor.

Table S3. Sensitivity analysis of the association between AIP and migraine with and without its components in the model.

| AIP Quartile | Original Model OR (95% CI) | Model Without TG & HDL-C OR (95% CI) | OR Change (%) |
| --- | --- | --- | --- |
| Q2 | 1.481 (1.105-1.984) | 1.501 (1.179-1.911) | +1.36% |
| Q3 | 1.366 (0.950-1.964) | 1.376 (1.040-1.822) | +0.77% |
| Q4 | 1.799 (1.228-2.637) | 1.711 (1.322-2.213) | -4.94% |

Note: OR: Odds ratio. CI: Confidence interval.
